# Consultations with Homœopaths

**Published:** 1858-07

**Authors:** 


CONSULTATIONS WITH HOMŒOPATHS.—Much remark has lately been elicited in the medical circles of London, as well as among the members of the profession generally in Great Britain, on account of the fact that several physicians and surgeons of high standing in the British metropolis are in the habit of meeting homœopaths in consultation. It appears that Mr. Fergusson is one of those who have rendered themselves particularly conspicuous in this respect. In a card, however, published a short time ago in one of the London Journals, he denies that he is in the *habit* of consulting with these men, but admits that he does not hesitate to meet them in all cases of emergency, on the ground that it would be contrary to the dictates of humanity to let a patient suffer simply because he was under the care of one of these practitioners. With all deference to the Queen's surgeon, we think him decidedly wrong in this opinion; nay, we go farther, and assert that any man who entertains such a view is totally ignorant of the fundamental principles of medical ethics and sadly unmindful of the duty he owes to his profession. What, forsooth, has suffering humanity to do in a matter of this kind? Is it not more probable that the great lever which moves to such a disgraceful violation of ethics is the expectation of a fat fee, or a dread to disregard the influence of the wealthy and influential? If his Grace, the Duke of Shetland, breaks his leg, and is fool enough to employ a homœopath until mortification has taken place, is it incumbent upon Mr. Fergusson to go to his relief when he is requested to do so, without declaring that he cannot meet his attendant, and that he will not recognize him in the case in any form or manner whatever? No practitioner is guilty of inhumanity who respects his own dignity and honor under such circumstances. His Grace need only dismiss the so-called doctor; the path is then clear, and any man may step in and occupy it. To consult with a homœopath is to place ourselves on a par with him; if the surgeon amputates a leg for him, and the after-treatment is confided to his care, it plainly implies that the surgeon has confidence in his skill and judgment, otherwise he would not allow the case to remain in his hands. What stronger endorsement could he give the quack? We are rejoiced to find that the profession of London has taken this matter in hand, and is determined to single out these friends of homœopathy. Let them take a bold stand upon the subject, and signally rebuke these men for the inconsistency and palpable impropriety of their conduct. The higher their position the more reason is there why they should be censured. An honorable physician should consider his profession as sacred as the person of his wife; he should no more think of coquetting with homœopathy than a virtuous husband should think of introducing a harlot into the domestic circle. It is an unclean thing, and should not be touched.

We believe this practice of consulting with homœopaths is not peculiar to our English brethren; indeed, we know it is not, for we have authentic information of the fact that the same offence has occasionally been perpetrated in this conntry. The instances, however, have been few, and we recollect no case where the outrage has been committed by a physician occupying a high professional position. What is more surprising still is that a State Medical Society, one of the oldest in America, should harbor homœopaths among its members. Can it be true, as is alleged in the May number of the St. Louis Medical and Surgical Journal, that the Massachusetts Medical Society is guilty of such an outrage? Our cotemporary asserts it as a "well-known fact," and justly adds that it is a blot on the fair name of this ancient and *once* honorable body.

